# Microstructural Characteristics and Material Failure Mechanism of SLM Ti-6Al-4V-Zn Alloy

**DOI:** 10.3390/ma16237341

**Published:** 2023-11-25

**Authors:** Yi-Jin Cheng, Fei-Yi Hung, Jun-Ren Zhao

**Affiliations:** Department of Materials Science and Engineering, National Cheng Kung University, Tainan 701, Taiwana2x346yz03@gmail.com (J.-R.Z.)

**Keywords:** Ti-6Al-4V, selective laser melting (SLM), mechanical properties, oxidation, phase transformation, particle erosion, 3D printing

## Abstract

This study focuses on the additive manufacturing technique of selective laser melting (SLM) to produce Ti-6Al-4V-Zn titanium alloy. The addition of zinc at 0.3 wt.% was investigated to improve the strength and ductility of SLM Ti-6Al-4V alloys. The microstructure and mechanical properties were analyzed using different vacuum heat treatment processes, with the 800-4-FC specimen exhibiting the most favorable overall mechanical properties. Additionally, zinc serves as a stabilizing element for the β phase, enhancing the resistance to particle erosion and corrosion impedance of Ti-6Al-4V-Zn alloy. Furthermore, the incorporation of trace amounts of Zn imparts improved impact toughness and stabilized high-temperature tensile mechanical properties to SLM Ti-6Al-4V-Zn alloy. The data obtained serve as valuable references for the application of SLM-64Ti.

## 1. Introduction

The advantage of additive manufacturing lies in its ability to customize products with complex shapes or unique structures while saving on tooling costs and material waste, thereby reducing production costs [1,2]. Currently, this technology has been widely applied in industries such as automotive, aerospace, and medical fields [2,3]. While additive manufacturing technology offers the above advantages, it is crucial to select appropriate processing technology and parameters to ensure the attainment of desirable properties [4,5]. In this study, Selective Laser Melting (SLM) was chosen as the additive manufacturing technology, using metal powder as the raw material [6]. The process involves melting the metal powder by high-energy laser scanning on a powder bed and rapidly cooling it to solidify into a specific shape [7,8]. The selected material is Ti-6Al-4V-Zn titanium alloy, which exhibits high strength-to-weight ratio, low elastic modulus, excellent corrosion resistance, and fatigue properties [9]. It is extensively used in industrial, aerospace, and medical applications [10], and is even employed in military applications [11]. With the development of modern industry, there is an increasing demand for titanium alloy products with higher dimensional accuracy and complex shapes. Therefore, the use of SLM as a forming technology for titanium alloys not only maintains the advantages of titanium alloys but also leverages the benefits of the SLM process, making it the preferred choice for industrial products. Furthermore, zinc metal is widely utilized across various industries. The addition of zinc elements and zinc coatings is prevalent in various fields, with galvanized coating being particularly common in industries due to its effective enhancement of corrosion and erosion resistance [12,13]. Moreover, the incorporation of trace amounts of zinc has been shown to enhance the strength, corrosion resistance, and processability of alloys [14,15,16]. Due to their similar hexagonal close-packed structures and electronegativity, the addition of zinc has minimal influence on the original Ti-6Al-4V alloy [17]. However, given the different atomic radii of Ti and Zn [18], it is important to note that the amount of added Zn should not be excessive.

In this study, zinc metal was chosen as an additive element in the Ti-6Al-4V titanium alloy, and an investigation into the microstructure and mechanical properties of the resulting Ti-6Al-4V-Zn titanium alloy was conducted [19]. Given that parts or equipment made of titanium alloys are susceptible to corrosion and damage at the metal/solution interface when exposed to corrosive media [20], we also conducted corrosion resistance investigations. Different vacuum heat treatments were applied to improve the properties of the Ti-6Al-4V-Zn alloy and to explore the effects of high temperature and oxygen content on its properties [21]. Furthermore, erosion wear is the phenomenon of gas- or liquid-driven particles impacting the surface of a material [22,23]. In numerous industrial applications of titanium alloys, including automotive and aerospace sectors, erosional wear caused by solid particles can lead to the failure of mechanical equipment and parts [23,24]. Despite the significance of particle erosion wear, there is a scarcity of studies on the erosion wear of SLM Ti-6Al-4V, let alone the SLM Ti-6Al-4V-Zn alloy investigated in this study. Therefore, it is crucial to establish particle erosion wear data and mechanisms for this alloy. Consequently, the changes in the structure, phase composition, and various properties of the alloy before and after particle erosion were examined [25,26]. Prior to this study, there were no related investigations on Ti-6Al-4V-Zn. Therefore, we conducted a comprehensive study on the SLM Ti-6Al-4V-Zn titanium alloy to obtain an in-depth understanding of the impact of zinc addition on SLM T-6Al-4V material properties. The generated data can offer valuable references for engineering or military applications of SLM-64Ti [27].

## 2. Materials and Methods

**Figure 1:**
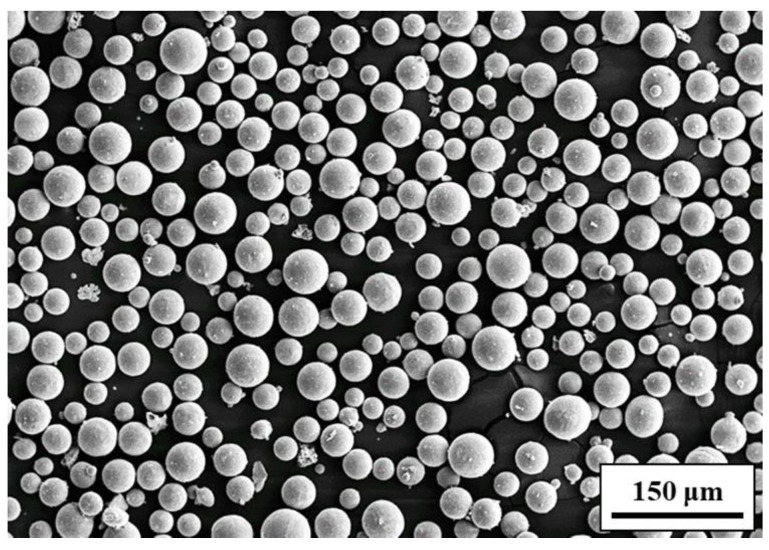
Morphology of Ti-6Al-4V-Zn powders.

**Figure 2:**
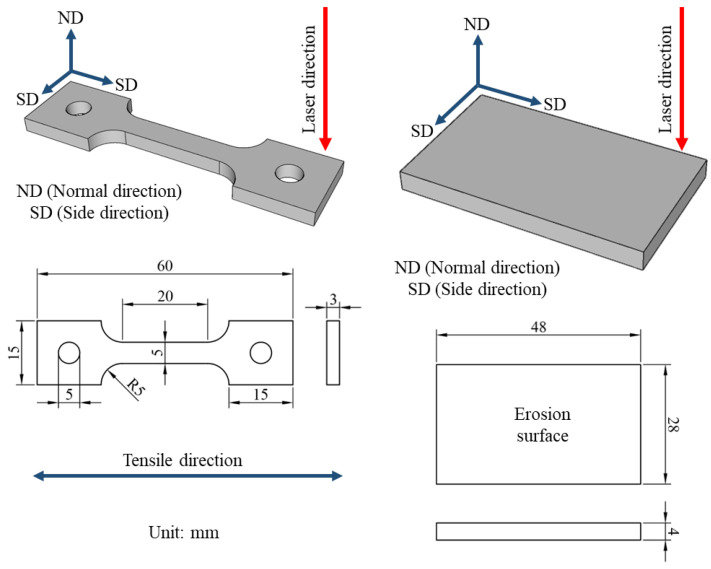
Schematic diagram of the dimensions for SLM processed tensile specimens and erosion test specimens.

The experimental material in this study is the Ti-6Al-4V-Zn alloy fabricated using selective laser melting (SLM). The powder, provided by Circle Metal Powder Co., Ltd. in Tainan, Taiwan, is illustrated in Figure 1 to showcase its morphology. The specimens were fabricated using an EOS M290 400 W machine manufactured by EOS in Krailling, Germany. The process parameters are presented in Table 1. The fabrication process took place in an inert gas (argon) atmosphere. The composition of the alloy elements is shown in Table 2, with a zinc content of 0.3 wt.%. According to the titanium-zinc alloy phase diagram [28], when the Zn content is 0.3 wt.%, α-Ti is the primary alloy phase rather than other titanium-zinc compounds. Additionally, the Zn content influences the phase transformation temperature of the titanium alloy. At this content, the phase transition temperature can be maintained between 800 and 900 °C. According to Chen et al. [29], the heat treatment at 850 °C leads to Ni_3_Ti precipitation and an increase in hardness, providing a basis for comparison with this study. Consisting of spherical particles with an average diameter of approximately 30 μm. Figure 2 illustrates the schematic and dimensions of the standard tensile specimens and erosion specimens obtained by wire-cutting the SLM Ti-6Al-4V-Zn alloy. The specimens include an SD surface perpendicular to the laser incidence direction and an ND surface parallel to the laser incidence direction. The dimensions of the tensile specimens are 20 mm in length, 5 mm in width, and 3 mm in thickness. The erosion specimens measure 48 mm in length, 28 mm in width, and 4 mm in thickness.

**Figure 3:**
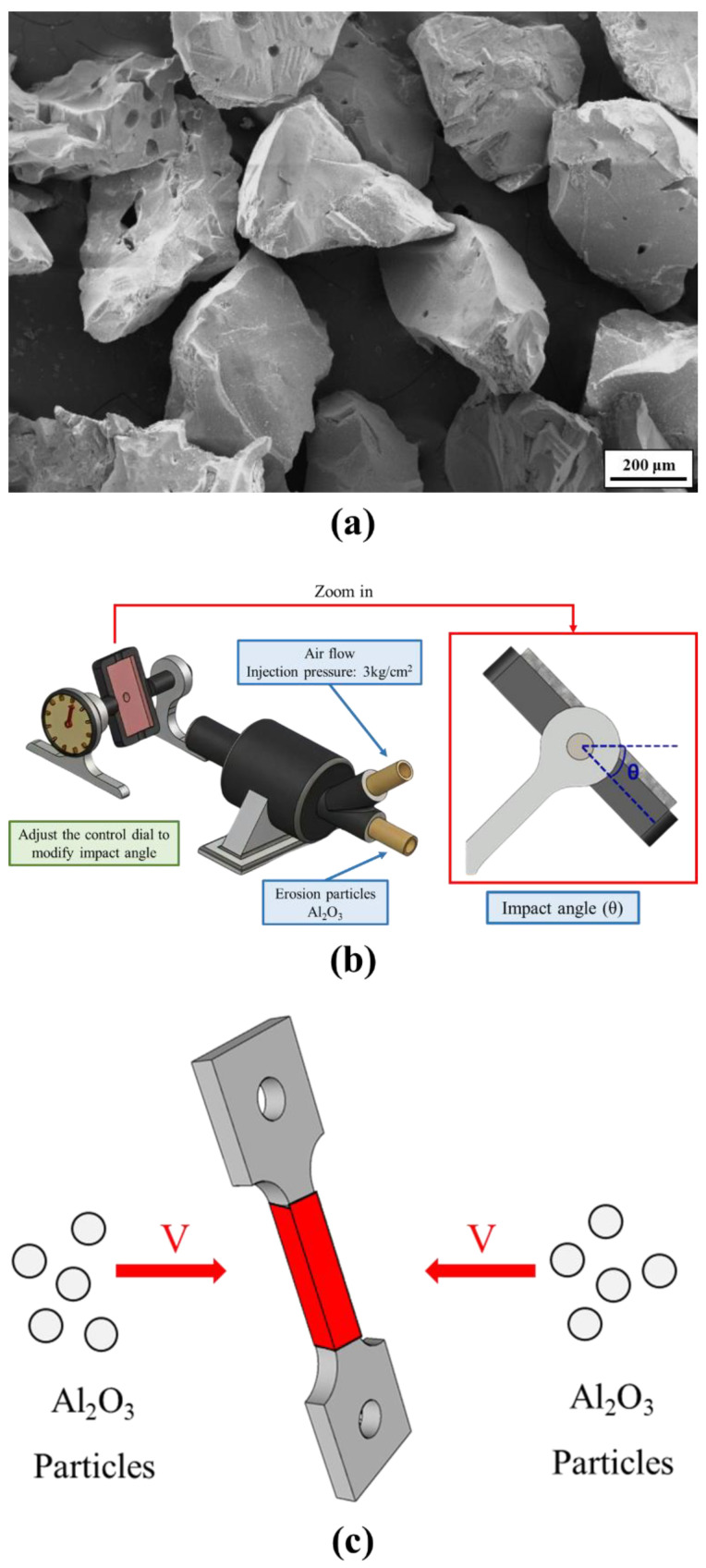
Particle erosion test: (**a**) morphology of erosion particles (Al_2_O_3_), (**b**) schematic diagram of the equipment, and (**c**) schematic diagram of double-sided erosion on parallel portion during tensile testing [30].

After subjecting the SLM Ti-6Al-4V-Zn tensile specimens to different vacuum heat treatment processes (800 °C and 900 °C), the microstructure and phase composition were analyzed using optical microscopy (OM, OLYMPUS BX41M-LED, Tokyo, Japan), scanning electron microscopy (SEM, HITACHI SU-5000, Hitachi, Tokyo, Japan), transmission electron microscopy (TEM, JEOL JEM-2100F, Hillsboro, OR, USA), and X-ray diffraction (XRD, Bruker AXS GmbH, Karlsruhe, Germany). The mechanical properties were evaluated using a Rockwell hardness tester (Rockwell hardness machine, RH, Mitutoyo, Kawasaki-shi, Japan), universal testing machine (HT-8336, Hung-Ta, Taichung, Taiwan), and impact testing machine (HT-8041A, Hung-Ta, Taichung, Taiwan). Fracture surfaces and sub-surfaces were examined, and the high-temperature effects, as well as the oxygen content effects, were investigated by considering variations in oxygen content among different batches of powder. Erosion experiments were conducted using alumina particles as shown in Figure 3a, and erosion properties of the SLM Ti-6Al-4V-Zn titanium alloy were analyzed using the schematic in Figure 3b [30]. The mechanical property differences after particle erosion were investigated by subjecting the tensile test specimens to double-sided erosion on the parallel portion, as shown in Figure 3c. Finally, the alloy polarization curves were measured and plotted to analyze the corrosion resistance properties. Based on multiple test results, application data for the SLM Ti-6Al-4V-Zn titanium alloy system were established.

## 3. Results and Discussion

**Figure 4:**
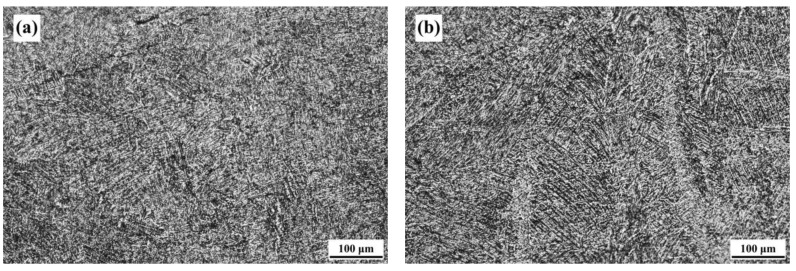
Microstructure of AS-printed material: (**a**) ND surface and (**b**) SD surface.

**Figure 5:**
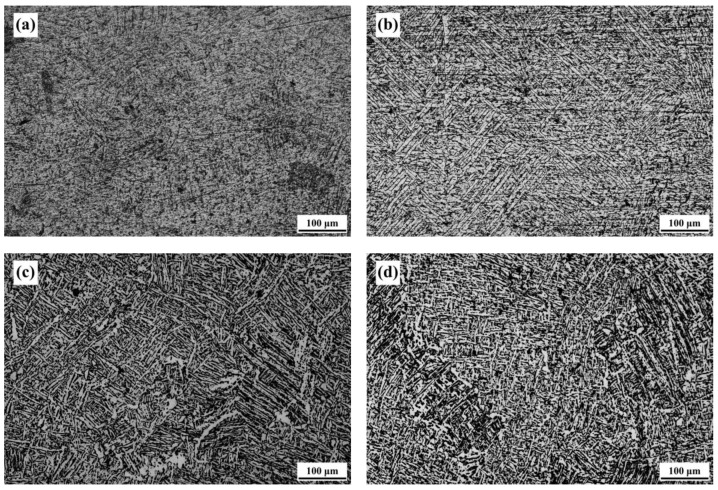
Microstructure of 800-FC material: (**a**) 1 h, (**b**) 2 h, (**c**) 4 h, and (**d**) 8 h.

**Figure 6:**
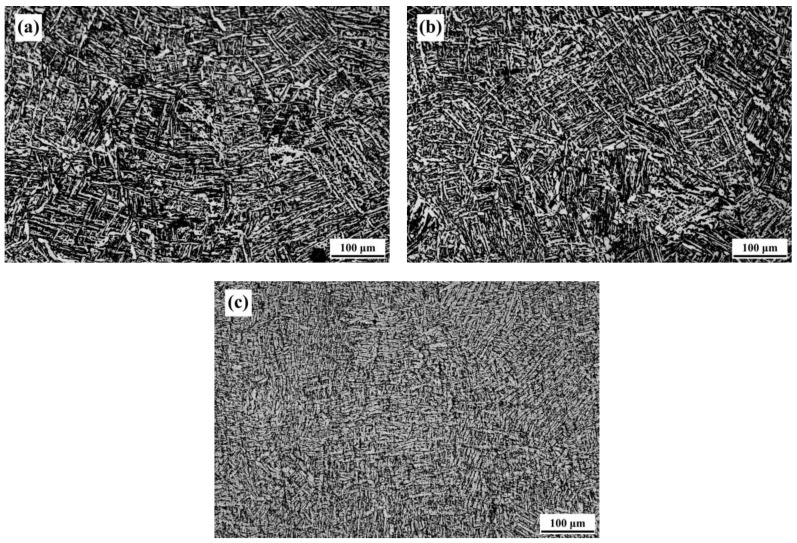
Microstructure of 900-FC material: (**a**) 1 h, (**b**) 2 h, and (**c**) 4 h.

In this study, the SLM Ti-6Al-4V-Zn titanium alloy, designated as the AS material, was used as the raw material. Figure 4 illustrates the microstructure of the ND and SD surfaces of the AS material. Both surfaces exhibit a needle-like microstructure, but the distribution on the ND surface is relatively more uniform. Therefore, the subsequent analysis of the heat-treated material focuses on the ND direction. The microstructure differs from the equiaxed primary β grain morphology observed in traditional cast Ti-6Al-4V alloy [31]. In comparison with the SLM Ti-6Al-4V alloy we previously studied [32], the needle-like phases in this study appear denser. Figure 5 shows the microstructure of the material after vacuum heat treatment at 800 °C. With increasing heat treatment time, the needle-like microstructure gradually transforms into a combination of needle-like and lamellar structures. The needle-like structure corresponds to the α phase, whereas the lamellar structure consists of alternating light-colored α phase and dark-colored β phase. Figure 6 displays the microstructure of the material after vacuum heat treatment at 900 °C, demonstrating an increased proportion of the lamellar structure with prolonged heat treatment time.

**Figure 7:**
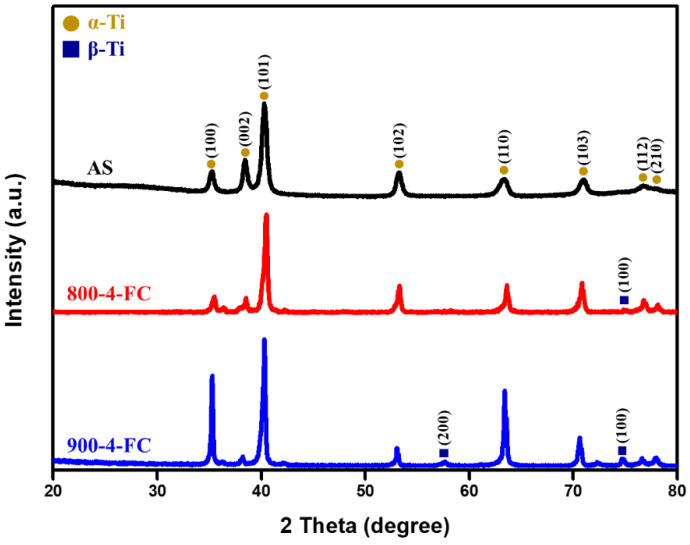
XRD analysis of AS, 800-4-FC, and 900-4-FC material.

**Figure 8:**
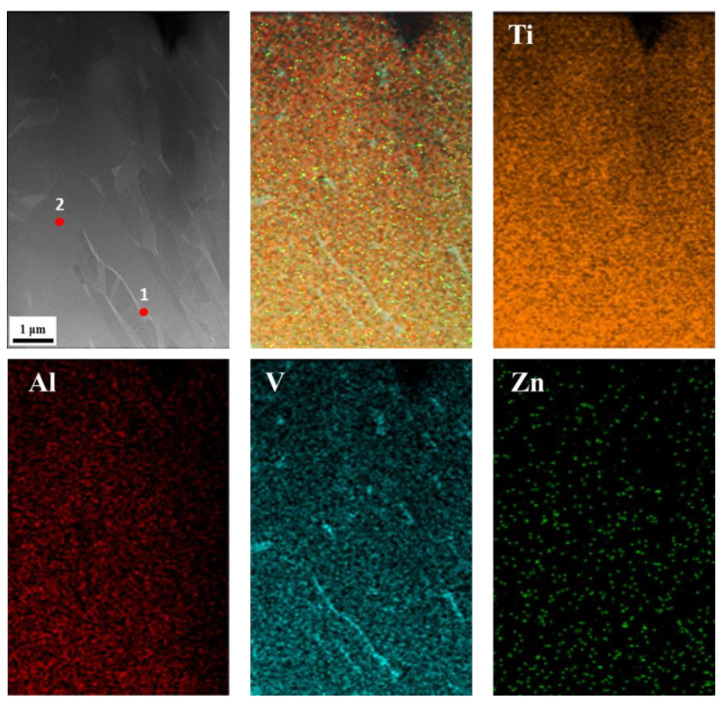
TEM specimen and elemental distribution of 800-4-FC material.

**Figure 9:**
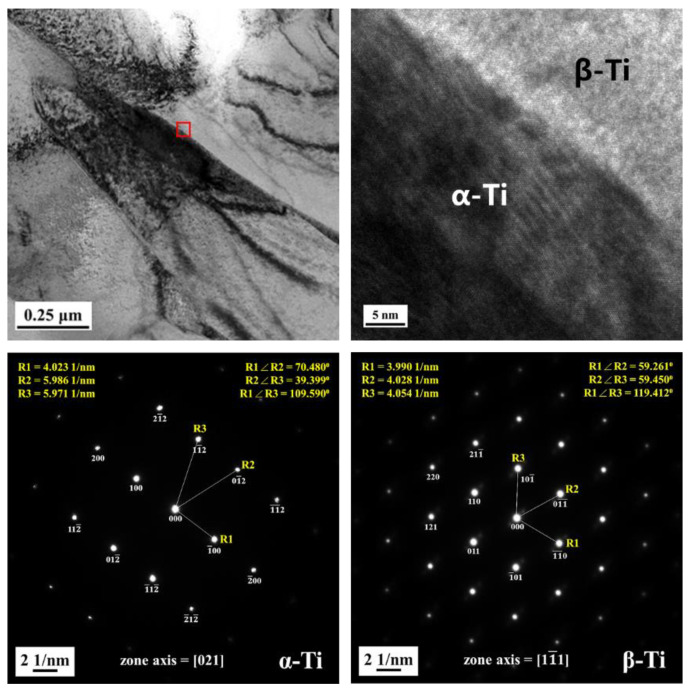
TEM phase identification analysis and atomic lattice with crystallographic axis analysis of 800-4-FC material.

Figure 7 shows the XRD diffraction analysis of AS material, 800-4-FC material, and 900-4-FC material. In the AS material, only α-phase peaks are observed. In contrast, both α-phase and β-phase peaks are detected in the 800-4-FC and 900-4-FC materials. However, no zinc-related peaks are detected in all three samples, indicating that the zinc content (only 0.3 wt.%) is relatively low. Figure 8 focuses on the TEM analysis of the 800-4-FC material (as it exhibits the best overall mechanical properties). It shows the uniform distribution of titanium and aluminum, with vanadium clustering in the light-colored regions. The 0.3 wt.% of zinc is primarily in a uniform solid solution, with only a very small amount accumulating at specific locations, as seen in Figure 8 at point 1. On the other hand, Figure 8 at point 2 only detects titanium, aluminum, and vanadium, without detecting any zinc. The elemental contents at these two points are shown in Table 3. Further analysis of point 1 reveals that the dark-colored regions in Figure 9 correspond to α phase titanium, while the light-colored regions correspond to β phase titanium. It can be inferred that the majority of zinc is uniformly dissolved in the titanium, with only a small portion of zinc accumulating around the β phase near the grain boundaries of the needle-like α phase. This zinc accumulation prevents the transformation of the needle-like α phase to the lamellar α + β phase during heat treatment, indicating that zinc addition helps stabilize the β phase.

**Figure 10:**
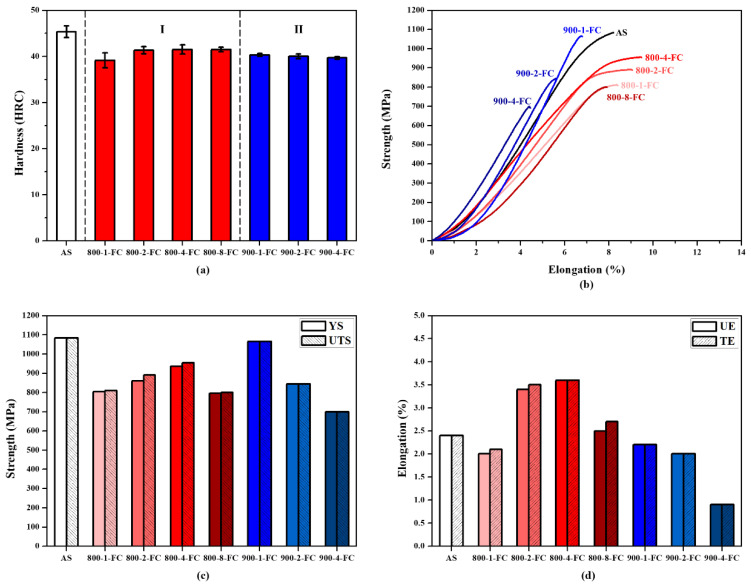
Mechanical properties of AS, 800-FC, and 900-FC material: (**a**) hardness, (**b**) tensile curve, (**c**) yield strength (YS) and ultimate tensile strength (UTS), and (**d**) uniform elongation (UE) and total elongation (TE).

**Figure 11:**
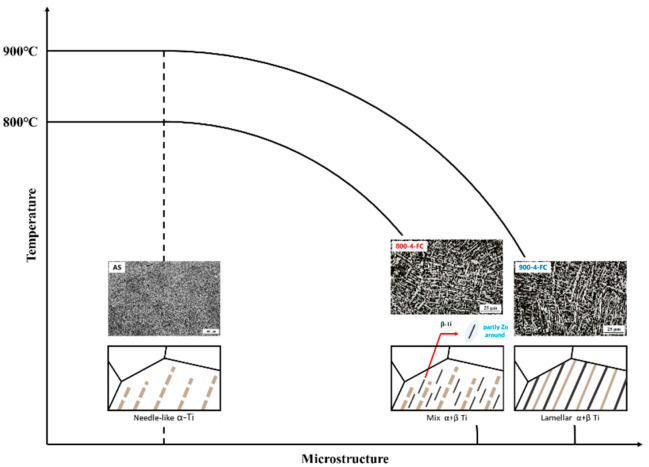
The mechanism of zinc addition on the tensile properties of AS, 800-FC, and 900-FC material.

**Figure 12:**
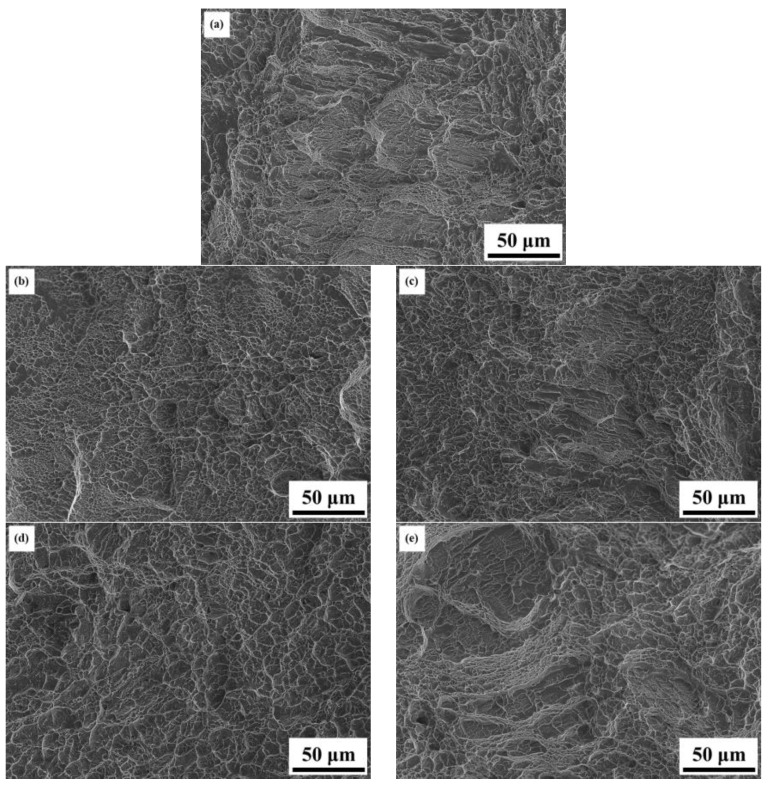
Room temperature tensile fracture surface morphology: (**a**) AS, (**b**) 800-1-FC, (**c**) 800-2-FC, (**d**) 800-4-FC, and (**e**) 800-8-FC.

Figure 10 presents the mechanical property data of the AS material and various heat-treated materials. In Figure 10a, a comparison of hardness is presented, showing that the AS material has a higher hardness (HRC 45) compared to all heat-treated materials (HRC 40). Figure 10b–d displays the tensile property data. The AS material exhibits the highest strength but poor ductility. The 800-4-FC material demonstrates the best overall tensile properties, combining both strength and ductility. The main reason for this is the coexistence of the needle-like α phase and lamellar α + β phase in the microstructure, as shown in Figure 11. The 800-4-FC material exhibits the optimal ratio of the needle-like to layered α + β phases, resulting in a combination of strength and ductility. Therefore, subsequent analysis mainly focuses on the AS and 800-4-FC materials. Figure 12 shows the fracture surfaces of the AS and 800-4-FC materials after tensile testing. Both fracture surfaces exhibit a combination of dimple-like structures and flat cleavage surfaces. The samples with higher ductility display a higher proportion of dimple-like structures, whereas flat cleavage surfaces dominate in the less ductile samples. Overall, when compared to traditional casting and SLM Ti-6Al-4V alloy [31,32], the failure mechanism of SLM Ti-6Al-4V-Zn is characterized by brittle dominance and poor ductility.

**Figure 13:**
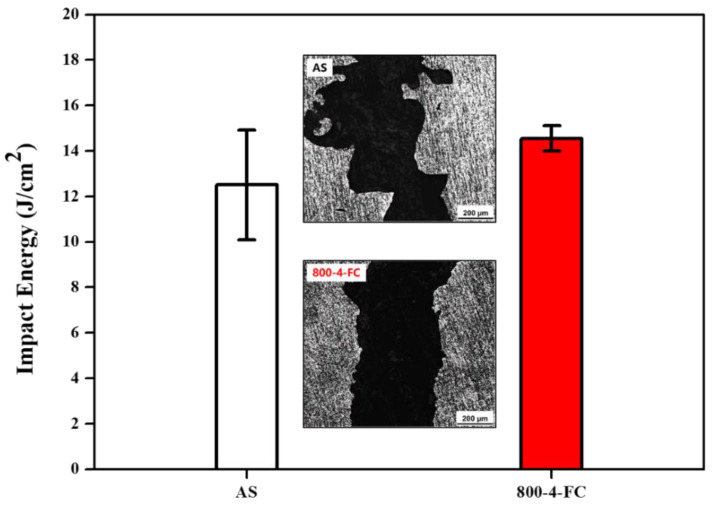
Impact energy and impact fracture sub-surface of AS and 800-4-FC material.

**Figure 14:**
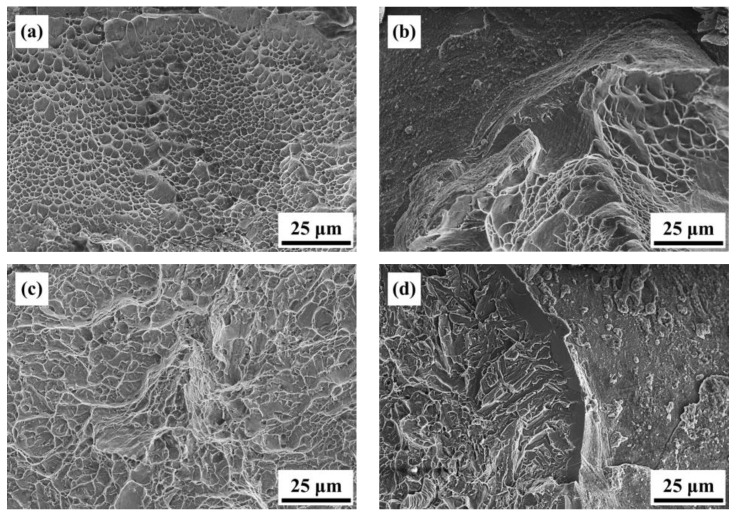
Impact fracture surfaces: (**a**) AS flat region, (**b**) AS cracked region, (**c**) 800-4-FC flat region, and (**d**) 800-4-FC cracked region.

Figure 13 presents the impact values and fracture sub-surfaces of the AS and 800-4-FC materials. The fracture sub-surface of the AS material shows numerous sharp fracture surfaces indicating crack propagation paths, with longer crack propagation distances. On the other hand, the fracture surface of the 800-4-FC material, (Figure 14), exhibits a layered structure with perpendicular fracture cracks, which effectively hinders crack propagation [32].

**Figure 15:**
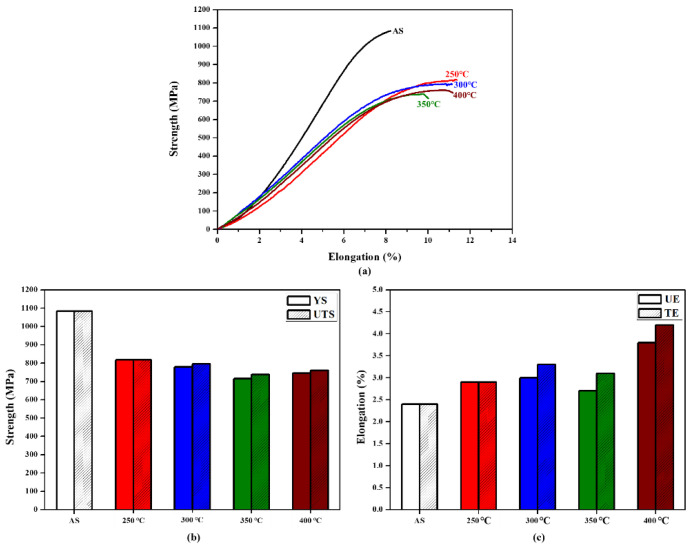
High-temperature tensile properties of AS material: (**a**) tensile curve, (**b**) yield strength and ultimate tensile strength, and (**c**) uniform elongation and total elongation.

**Figure 16:**
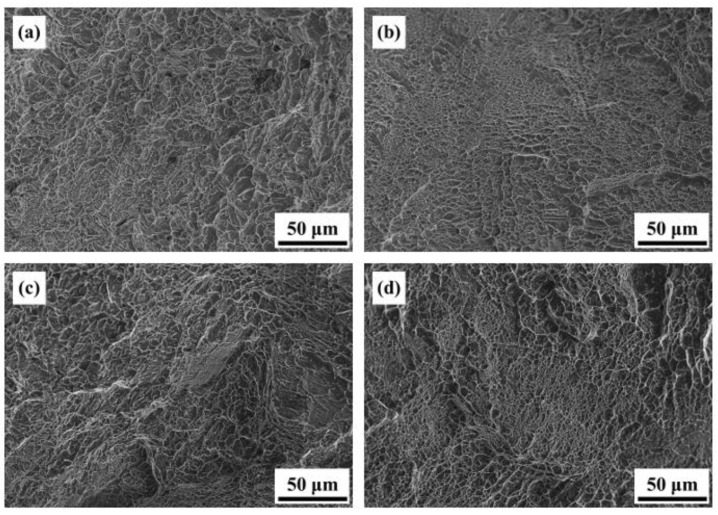
High-temperature tensile fracture surface morphology of AS material: (**a**) 250 °C, (**b**) 300 °C, (**c**) 350 °C, and (**d**) 400 °C.

Figure 15 displays the high-temperature tensile data of the AS material in the range of 250 °C to 400 °C. It can be observed that an increase in temperature leads to a decrease in strength and an improvement in ductility. Overall, the material still retains its applicability. Figure 16 illustrates the fracture surfaces of the AS material under high-temperature tensile testing. The proportion of dimple-like structures, which represents the ductile behavior of the material, increases with temperature. The size of the dimples is inversely proportional to the ductility. Moreover, the increase in temperature does not result in significant high-temperature oxidation effects in the AS material. The differences in fracture surfaces at different temperatures are minimal, indicating that the SLM Ti-6Al-4V-Zn titanium alloy can maintain stability at high temperatures [32].

**Figure 17:**
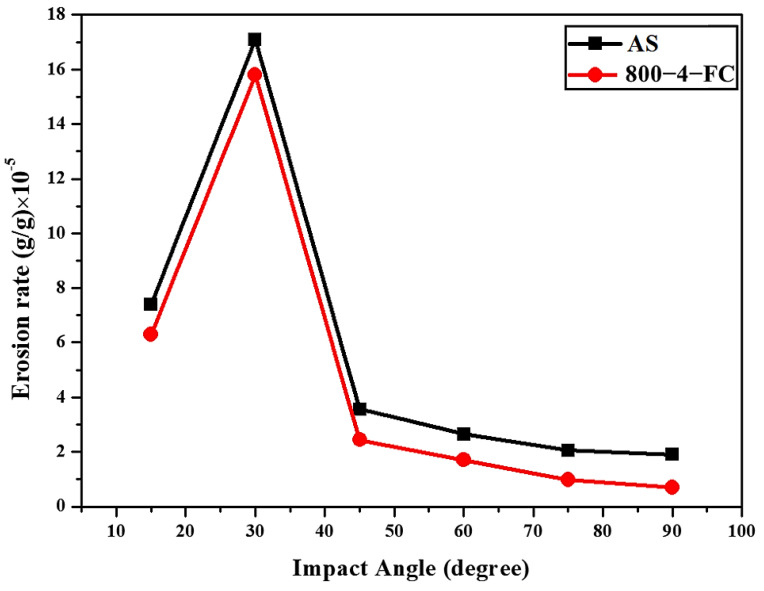
Erosion rates of AS and 800−4−FC material.

Figure 17 presents the particle erosion weight loss rates of the AS material and the 800-4-FC material. Both materials exhibit peak erosion rates at 30° and the lowest rates at 90°. The erosion behavior in both cases is dominated by ductile deformation [30]. Moreover, for any erosion angle, the 800-4-FC material demonstrates lower erosion rates compared to the AS material. This indicates that the 800-4-FC material, which consists of a combination of the needle-like α phase and lamellar α + β phase, exhibits superior erosion resistance compared to the AS material, which consists only of the needle-like α phase. Furthermore, the erosion rates of SLM Ti-6Al-4V gradually decrease from 30° to 90°.

**Figure 18:**
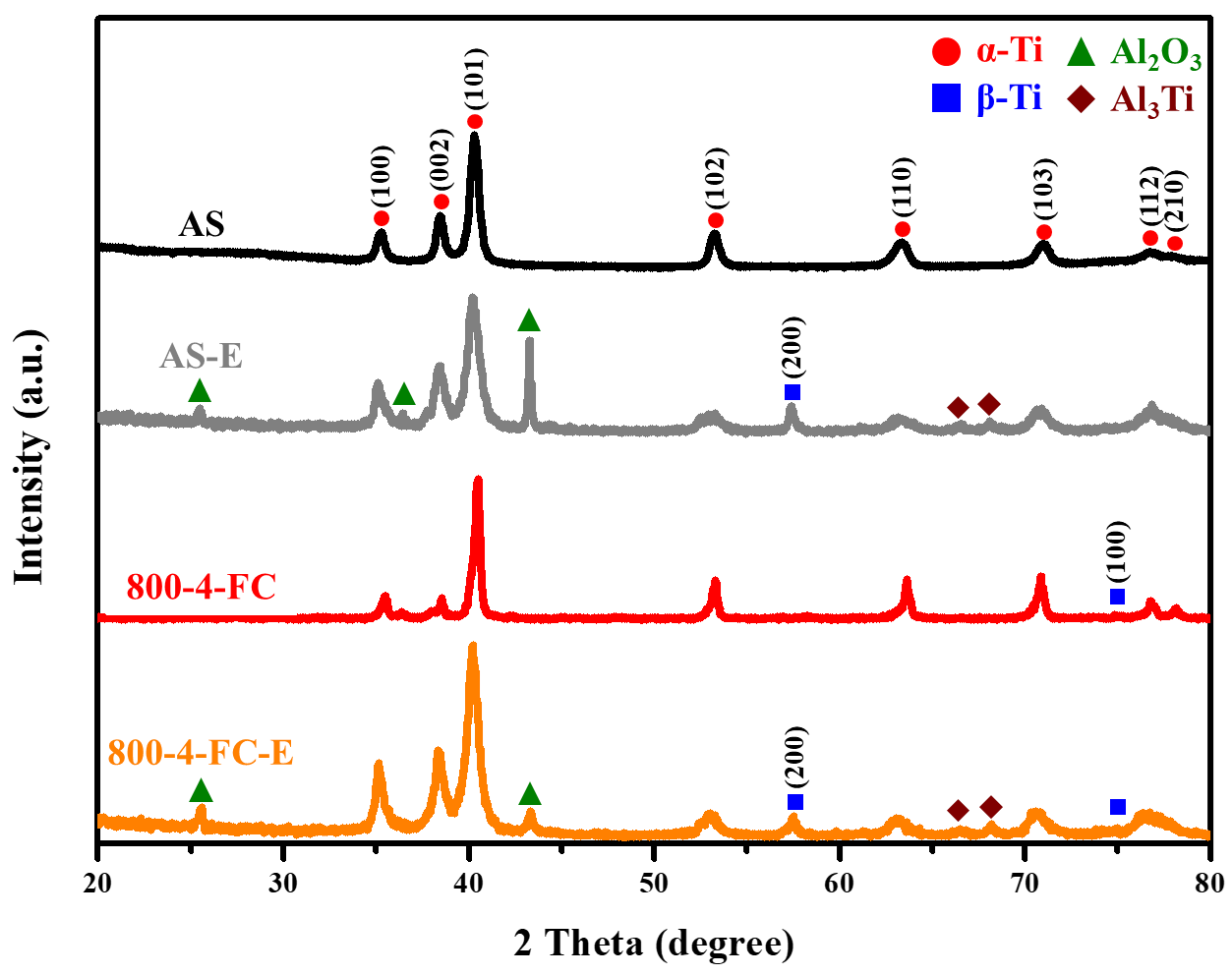
XRD analysis of AS and 800-4-FC material before and after particle erosion.

Figure 18 illustrates the XRD analysis of the AS material and the 800-4-FC material before and after particle erosion at 90°. The appearance of peaks corresponding to Al_2_O_3_ and Al_3_Ti can be observed. This is attributed to the reaction between the sample surface and the eroded particles during the particle erosion process, as the surface temperature of the sample can reach up to 500 °C [32]. It is also possible that residual eroded particles remain on the sample surface, leading to the formation of these new phases. Furthermore, the peaks corresponding to Al_2_O_3_ and Al_3_Ti are more pronounced in the AS-E material compared to the 800-4-FC-E material. This is attributed to the transformation of the microstructure into a combination of needle-like and lamellar structures after heat treatment, which effectively prevents the impact of particles. This mechanism has been reported in our previous study [32].

**Figure 19:**
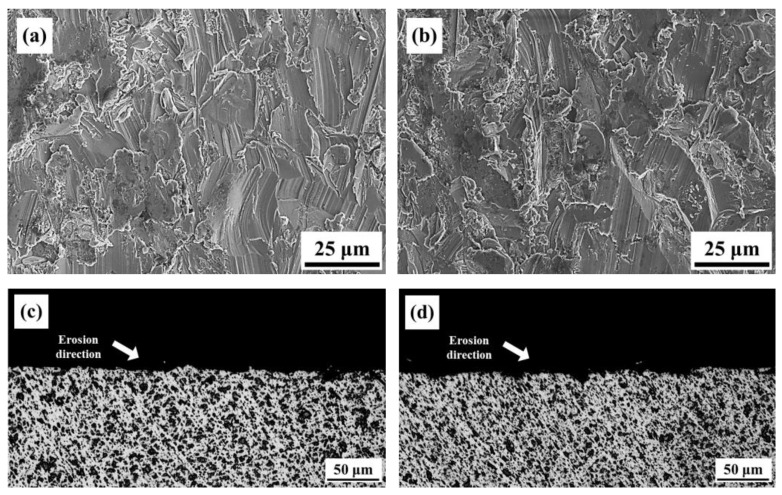
Surface and sub-surface morphology after 30° erosion: (**a**) AS surface, (**b**) 800-4-FC surface, (**c**) AS sub-surface, and (**d**) 800-4-FC sub-surface.

**Figure 20:**
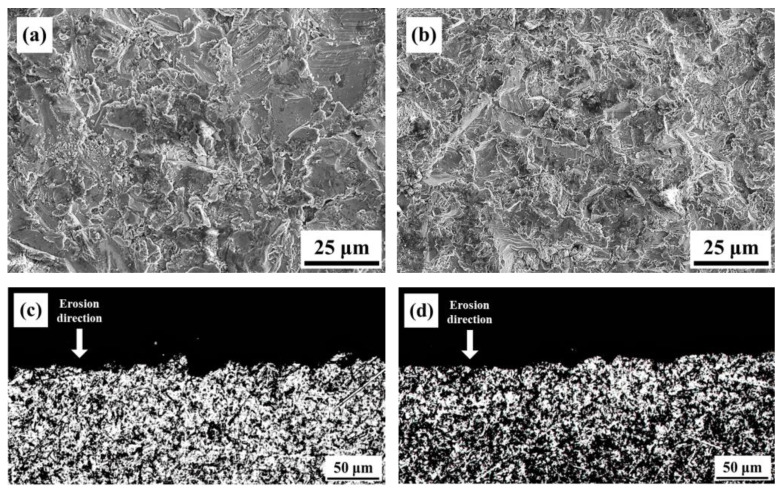
Surface and sub-surface morphology after 90° erosion: (**a**) AS surface, (**b**) 800-4-FC surface, (**c**) AS sub-surface, and (**d**) 800-4-FC sub-surface.

Observations were made on the eroded surfaces at 30° and 90° angles. After erosion at 30°, distinct and directional scratches were observed on the surface of the specimens, and localized plowing features were also present, as shown in Figure 19a,b. A comparison between the two angles revealed that the 800-4-FC material exhibited fewer surface scratches and plowing features. Similar observations were made on the sub-surface after erosion at 30°, as depicted in Figure 19c,d, with the sub-surface of the 800-4-FC material appearing relatively smoother, whereas the AS material exhibited more pronounced damage. For erosion at 90°, the eroded surfaces and sub-surfaces also exhibited less damage in the case of the 800-4-FC material. A comparison of the erosion surfaces in Figure 20a,b showed that the AS material exhibited more pits and larger scratched areas. On the other hand, in Figure 20c,d, which represents the sub-surfaces after 90° erosion, the AS material had narrower and deeper pits, whereas the 800-4-FC material had shallower and wider pits. The observations from Figure 19 and Figure 20 collectively show that the 800-4-FC material possesses superior erosion resistance properties [33].

**Figure 21:**
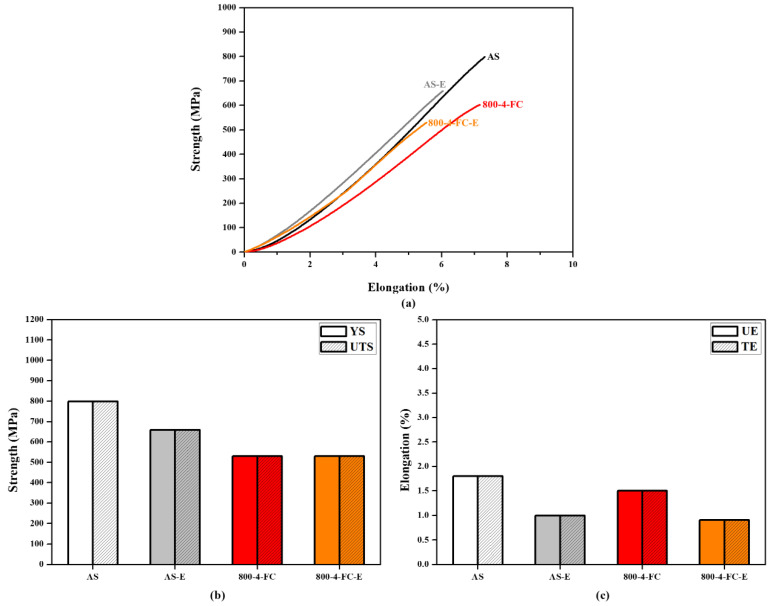
Tensile properties before and after particle erosion in AS and 800-4-FC material: (**a**) tensile curve, (**b**) yield strength and ultimate tensile strength, and (**c**) uniform elongation and total elongation.

**Figure 22:**
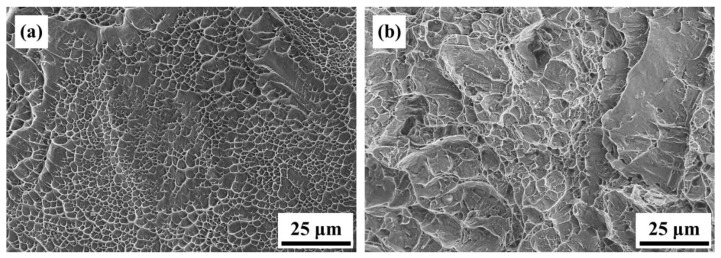
Tensile fracture surfaces after particle erosion: (**a**) AS and (**b**) 800-4-FC.

Figure 21 illustrates the differences in tensile properties between the AS and 800-4-FC materials before and after particle erosion. Compared to the AS material, the 800-4-FC material exhibited less deterioration in tensile properties after erosion, although both materials experienced a decrease in tensile strength following the erosion process. Figure 22 displays the tensile fracture surfaces of the AS and 800-4-FC materials after erosion. The 800-4-FC material exhibited predominantly cleavage fracture, whereas the AS material showed only the localized regions of ductile dimples. The reduction in tensile properties can be attributed to the formation of new phases at high temperatures during particle erosion and the damage caused to the specimens during the erosion process.

**Figure 23:**
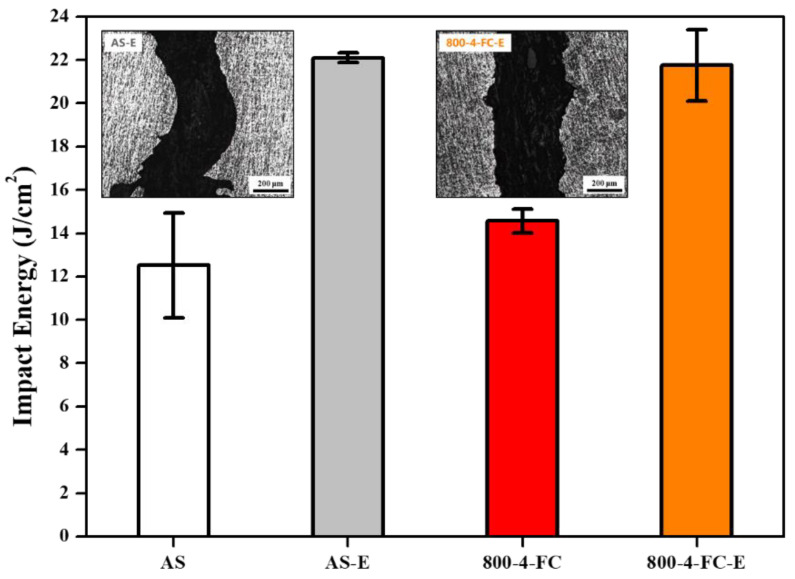
Impact energy and impact fracture sub-surfaces before and after particle erosion of AS and 800-4-FC material.

Figure 23 presents the differences in impact values and impact fracture sub-surfaces of the AS and 800-4-FC materials before and after particle erosion. It can be observed that the impact values increased after erosion, but there were no significant differences in the crack propagation path compared to before erosion. The increase in energy absorption is attributed to the surface work hardening and the formation of a surface layer induced by particle erosion, which helps to absorb and disperse the impact energy [34].

**Figure 24:**
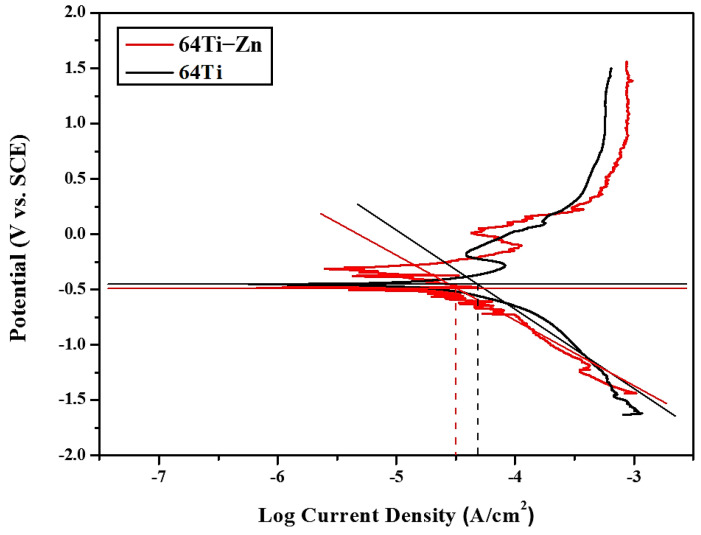
Polarization curves of SLM Ti−6Al−4V AS material and SLM Ti−6Al−4V−Zn AS material.

Figure 24 compares the polarization curves of SLM Ti-6Al-4V-Zn AS material and SLM Ti-6Al-4V AS material [35]. Zinc itself is a corrosion-resistant metal, and according to our TEM results (Figure 8 and Figure 9), most of the zinc in SLM Ti-6Al-4V-Zn is uniformly dissolved in titanium. Consequently, it effectively enhances corrosion resistance, resulting in a lower corrosion current density for SLM Ti-6Al-4V-Zn alloy (0.029 A/cm^2)^ compared to SLM Ti-6Al-4V alloy (0.039 A/cm^2^).

Based on several tests evaluating practical applicability and analyzing mechanisms, the 800-4-FC material demonstrates the best overall mechanical properties. It exhibits a combination of strength and ductility, maintains stability in high-temperature environments, and improves erosion and corrosion resistance compared to other materials.

## 4. Limitations

The influence of the phase transformation layer on the tensile and impact properties after particle erosion can be further studied, including the thickness, phase structure, and high-temperature stability of the phase transformation layer.

## 5. Conclusions

The microstructural characteristics of SLM Ti-6Al-4V-Zn exhibit a uniform distribution of needle-shaped α phase in the ND plane. With increasing heat treatment temperature, this structure gradually transforms into a layered structure of α + β phases. Zinc in the SLM Ti-6Al-4V-Zn titanium alloy is primarily distributed in a homogeneous solid solution within the titanium matrix, with only a small portion clustering and inhibiting the phase transformation of the needle-shaped α phase. Therefore, the addition of zinc effectively stabilizes the β phase, and the incorporation of trace amounts of zinc does not impact its industrial applicability.

The vacuum heat treatment at 800 °C for 4 h followed by furnace cooling (800-4-FC) enhances ductility while maintaining strength in SLM Ti-6Al-4V-Zn. This is attributed to the coexistence of needle-shaped α phase and layered α + β phase in the optimal proportion, resulting in the best combination of tensile mechanical properties and impact energy. Additionally, the heat treatment improves the high-temperature oxidation resistance of the alloy, thereby enabling its industrial applicability even at elevated temperatures.

Particle erosion leads to the formation of new phases on the surface of SLM Ti-6Al-4V-Zn specimens due to high-temperature phase transformation and the reaction with eroded particles. Particle erosion induces surface work hardening, resulting in the formation of a hardened layer and improved impact toughness. The industrial applicability of SLM Ti-6Al-4V-Zn is enhanced by the addition of zinc, which improves its resistance to erosion and corrosion. Furthermore, its application performance is further enhanced via heat treatment.

## Figures and Tables

**Table 1:** Parameters of the process for SLM Ti-6Al-4V-Zn alloy.

Particle Size	Laser Power	Laser Radius	Scanning Velocity	Layer Thickness
15–53 μm	170 W	35 μm	1000 mm/s	60 μm

**Table 2:** Chemical composition of SLM Ti-6Al-4V-Zn alloy (wt.%).

Element	Al	V	Zn	O	C	N	Ti
Wt.%	5.7	3.7	0.3	0.2	0.1	0.1	Bal.

**Table 3:** TEM elemental analysis of the 800-4-FC specimen.

Element		Ti	Al	V	Zn
**at.%**	1	75.27	3.58	21.09	0.07
	2	87.26	9.42	3.32	0.00

## Data Availability

Datais contained within the article.

## References

[1] Jiang J. (2023). A survey of machine learning in additive manufacturing technologies. Int. J. Comput. Integr. Manuf..

[2] Nazir A., Gokcekaya O., Billah K.M.M., Ertugrul O., Jiang J., Sun J., Hussain S. (2023). Multi-material additive manufacturing: A systematic review of design, properties, applications, challenges, and 3D Printing of materials and cellular metamaterials. Mater. Des..

[3] Peters M., Kumpfert J., Ward C.H., Leyens C. (2003). Titanium alloys for aerospace applications. Adv. Eng. Mater..

[4] Niu X., Zhu S.P., He J.C., Liao D., Correia J.A., Berto F., Wang Q. (2022). Defect tolerant fatigue assessment of AM materials: Size effect and probabilistic prospects. Int. J. Fatigue.

[5] Fang J.X., Wang J.X., Wang Y.J., He H.T., Zhang D.B., Cao Y. (2022). Microstructure evolution and deformation behavior during stretching of a compositionally inhomogeneous TWIP-TRIP cantor-like alloy by laser powder deposition. Mater. Sci. Eng. A.

[6] Nandhakumar R., Venkatesan K. (2023). A process parameters review on Selective laser melting-based additive manufacturing of Single and Multi-Material: Microstructure, Properties, and machinability aspects. Mater. Today Commun..

[7] Yap C.Y., Chua C.K., Dong Z.L., Liu Z.H., Zhang D.Q., Loh L.E., Sing S.L. (2015). Review of selective laser melting: Materials and applications. Appl. Phys. Rev..

[8] Peng J., Li J., Liu B., Wang J., Chen H., Feng H., Zeng X., Duan H., Cao Y., He J. (2023). Formation process and mechanical properties in selective laser melted multi-principal-element alloys. J. Mater. Sci. Technol..

[9] Liu S., Shin Y.C. (2019). Additive manufacturing of Ti6Al4V alloy: A review. Mater. Des..

[10] Wang Z., Zhou W., Luo K., Lu H., Lu J. (2023). Strengthening mechanism in thermomechanical fatigue properties of Ti6Al4V titanium alloy by laser shock peening. Int. J. Fatigue.

[11] Zochowski P., Bajkowski M., Grygoruk R., Magier M., Burian W., Pyka D., Jamroziak M.B.K. (2021). Ballistic impact resistance of bulletproof vest inserts containing printed titanium structures. Metals.

[12] Yao H.L., Hu X.Z., Yi Z.H., Xia J., Tu X.Y., Li S.B., Yu B., Zhang M.X., Bai X.B., Chen Q.Y. (2021). Microstructure and improved anti-corrosion properties of cold-sprayed Zn coatings fabricated by post shot-peening process. Surf. Coat. Technol..

[13] Maniam K.K., Paul S. (2021). Corrosion performance of electrodeposited zinc and zinc-alloy coatings in marine environment. Corros. Mater. Degrad..

[14] Pola A., Tocci M., Goodwin F.E. (2020). Review of microstructures and properties of zinc alloys. Metals.

[15] Arun S., Lim B.-S., Ahn S.-G., Choe H.-C. (2023). Osteoconductive element-doped, porous, and low-elastic-modulus duplex coatings on a Ti-6Al-4V alloy: A hybrid coating system for accelerating cell growth. J. Alloys Compd..

[16] Liu W., Zhao Y., Zhang Y., Shuai C., Chen L., Huang Z., Hou H. (2023). Deformation-induced dynamic precipitation of 14H-LPSO structure and its effect on dynamic recrystallization in hot-extruded Mg-Y-Zn alloys. Int. J. Plast..

[17] Zhang Y., Chen X., Jayalakshmi S., Singh R.A., Deev V.B., Prusov E.S. (2021). Factors determining solid solution phase formation and stability in CoCrFeNiX_0.4_ (X = Al, Nb, Ta) high entropy alloys fabricated by powder plasma arc additive manufacturing. J. Alloys Compd..

[18] Xu C., Wu Q., Hua Y., Li J. (2014). The electrodeposition of Zn-Ti alloys from ZnCl_2_-urea deep eutectic solvent. J. Solid State Electrochem..

[19] Saidi R., Ashrafizadeh F., Raeissi K., Kharaziha M. (2020). Electrochemical aspects of zinc oxide electrodeposition on Ti6Al4V alloy. Surf. Coat. Technol..

[20] Wang N.X., Wang Y.S., Zheng K., Zhi J.Q., Zhou B., Wu Y.X., Xue X.P., Ma Y., Cheng F., Gao J. (2023). Achieving CVD diamond films on Mo_0. 5_ (TiZrTaW) _0.5_ highly concentrated alloy for ultrastrong corrosion resistance. Surf. Coat. Technol..

[21] Gogia A. (2005). High-temperature titanium alloys. Def. Sci. J..

[22] Finnie I. (1973). Some observations on the erosion of ductile metals. Wear.

[23] Dai W.S., Chen L.H., Lui T.S. (2000). A study on SiO_2_ particle erosion of flake graphite and spheroidal graphite cast irons. Wear.

[24] Cai F., Huang X., Yang Q. (2015). Mechanical properties, sliding wear and solid particle erosion behaviors of plasma enhanced magnetron sputtering CrSiCN coating systems. Wear.

[25] Khoddami A., Salimi-Majd D., Mohammadi B. (2019). Finite element and experimental investigation of multiple solid particle erosion on Ti-6Al-4V titanium alloy coated by multilayer wear-resistant coating. Surf. Coat. Technol..

[26] Saidi R., Raeissi K., Ashrafizadeh F., Kharaziha M. (2021). The effect of zinc oxide coating morphology on corrosion performance of Ti-6Al-4V alloys. J. Alloys Compd..

[27] Lv Z., Li H., Che L., Chen S., Zhang P., He J., Wu Z., Niu S., Li X. (2023). Effects of HIP Process Parameters on Microstructure and Mechanical Properties of Ti-6Al-4V Fabricated by SLM. Metals.

[28] Okamoto H. (2008). Ti-Zn (titanium-zinc). J. Phase Equilibria Diffus..

[29] Chen Y., Sun S., Zhang T., Zhou X., Li S.S. (2020). Effects of post-weld heat treatment on the microstructure and mechanical properties of laser-welded NiTi/304SS joint with Ni filler. Mater. Sci. Eng. A.

[30] Zhao J.R., Hung F.Y., Lui T.S. (2019). Particle erosion induced phase transformation of different matrix microstructures of powder bed fusion Ti-6Al-4V alloy flakes. Metals.

[31] Elshaer R.N., Ibrahim K.M. (2023). Study of microstructure; mechanical properties, and corrosion behavior of as-cast Ni-Ti and Ti-6Al-4V alloys. J. Mater. Eng. Perform..

[32] Zhao J.R., Hung F.Y., Lui T.S., Wu Y.L. (2019). The relationship of fracture mechanism between high temperature tensile mechanical properties and particle erosion resistance of selective laser melting Ti-6Al-4V alloy. Metals.

[33] Huang B.C., Chang K.C., Hung F.Y., Microstructure S.O. (2021). Mechanical Properties and Erosion Characteristics of Al-Si Alloy Manufactured by Continuous Casting Direct Rolling Process. Appl. Sci..

[34] Chen H., Zhao D., Wang Q., Qiang Y., Qi J. (2017). Effects of impact energy on the wear resistance and work hardening mechanism of medium manganese austenitic steel. Friction.

[35] Zhang Q., Duan B., Zhang Z., Wang J., Si C. (2021). Effect of ultrasonic shot peening on microstructure evolution and corrosion resistance of selective laser melted Ti–6Al–4V alloy. J. Mater. Res. Technol..

